# Molecular diagnosis of bovine tuberculosis on postmortem carcasses during routine meat inspection in Benin: GeneXpert^®^ testing to improve diagnostic scheme

**DOI:** 10.14202/vetworld.2022.2506-2510

**Published:** 2022-10-31

**Authors:** Cyrille K. Boko, Ange-Régis Zoclanclounon, Camus M. Adoligbe, Hebert Dedehouanou, Marguéritte M’Po, Samuel Mantip, Souaïbou Farougou

**Affiliations:** 1 Research Unit in Transmissible Diseases, Polytechnic School of Abomey-Calavi, University of Abomey-Calavi, 01BP 2009, Cotonou, Benin; 2 National Center of TB Control, Cotonou, Benin; 3 Virology Division, National Veterinary Research Institute, PMB 0001, Vom, Nigeria

**Keywords:** genomic amplification, non-tuberculosis mycobacteria, one health, polymerase chain reaction accuracy, zoonotic tuberculosis

## Abstract

**Background and Aim:**

Bovine tuberculosis (TB) is a zoonotic disease of major public health importance, particularly in African countries, where control measures are limited or largely not applied. This study aimed to determine the accuracy of the currently used bovine TB diagnostic method at slaughterhouses in Benin; this is to contribute to the betterment and improvement in the epidemiological surveillance of the disease in the country.

**Materials and Methods:**

A total of 40 tissue samples were collected from meat/viscera (lung, liver, heart, kidney, and the gastro-intestinal tract tissues) at Cotonou slaughterhouses from ruminants suspected to be infected with bovine TB during routine meat inspection. The collected samples were analyzed using GeneXpert testing technique as a reference method.

**Results:**

Twenty-six samples tested positive out of the 40 suspected tissue samples collected by GeneXpert diagnostic technique; this shows the limitation of the routine meat inspection in detecting bovine TB as currently performed in Benin.

**Conclusion:**

The outcome of the use of the molecular technique, therefore, supports the importance of the use of a molecular tool alongside the routine meat inspection for a better understanding of the epidemiology of bovine TB in Benin. However, more robust technical and policy efforts are needed for a sustainable implementation of such a strategy.

## Introduction

Zoonoses are pathologies or contagious diseases whose agents are naturally transmitted from animals to humans, and vice versa. They account for more than 61% of emerging diseases affecting the world today [1, 2]. These diseases are fast spreading mainly as a result of an increased human activities. The people who are prone to zoonoses include herders, veterinarians, foresters, and farmers. The zoonoses with a very significant health impact in the world include rabies, avian influenza, leishmaniasis, brucellosis, and bovine tuberculosis (TB) [3]. Bovine TB is a major zoonosis with the risk of interspecies contamination. It is mainly caused by *Mycobacterium bovis* and it can induce respiratory disorders in both cattle and humans [4].

Bovine TB is classified in a list B of communicable diseases of public health and socio-economic importance [5]. Thanks to the implementation of an efficient and regular surveillance system which enables many developed countries to successfully eradicate bovine TB [6, 7]. Unfortunately, in developing countries, including Benin, bovine TB is still endemic due to the lack of epidemiologically adapted control measures [8]. In Benin, bovine TB diagnosis is essentially based on the physical detection of bovine TB-like lesions during a routine veterinary inspection at the abattoir, and bovine TB detection always constitutes the main reason for meat and viscera condemnation during postmortem [9]. However, bovine TB-like lesions are not all pathognomonic. Hence, bovine TB detection based on bovine TB-like lesions may lack specificity and resulting data may not be accurate.

The aim of this study was to test a more sensitive and much more specific tool such as the real-time polymerase chain reaction (PCR) assay (GeneXpert^®^, Cepheid, France) as an alternative to the routine meat inspection to have a better view of the current incidence of bovine TB in cattle at slaughterhouses. The Xpert *M. bovis*/Rifampicin assay is a new test that is revolutionizing TB control and prevention by contributing as a rapid diagnostic test kit for TB disease and drug resistance. The test simultaneously detects *M. bovis* complex and resistance to rifampicin in <2 h [10]. In comparison, standard cultures can take 2−6 weeks for Mycobacterium to grow and the conventional indirect drug resistance tests can prolong the period of treatment by 3 more weeks.

## Materials and Methods

### Ethical approval

All the samples used for this study were issued from animals analyzed within an official context. No purposeful killing of animals was performed for this study. All samplings were in complete agreement with national regulations. Therefore, in that regard, no ethical approval was necessary.

### Study period and location

The study was conducted from May to August 2020. This study was carried out in the main slaughterhouse of Benin (Cotonou slaughterhouse), Department of Littoral. The analysis was conducted in the National TB control center (PNT) and the Unit Research on Transmissible Disease (URMaT).

### Sampling

Samples collected during the period of our study presented macroscopic bovine TB-like lesions at routine abattoir inspection. In total, 40 tissue samples of approximately 10 g each were collected from condemned meat or viscera parts using a scalpel blade and holder. Samples were transported to the laboratory in cold storage condition and stored in 4°C refrigerator before laboratory analysis [11].

### Fluorescent microscopy

Briefly, the microscopic diagnosis was based on auramine staining following the protocol established by Vrain [12].

### Quantitative PCR (GeneXpert^®^ test)

The GeneXpert^®^ test detects DNA sequences specific for *Mycobacterium tuberculosis* complex and rifampicin resistance by PCR. It is based on the Cepheid GeneXpert system, a rapid, simple-to-use nucleic acid amplification test [13]. The process amplifies DNA sequences in a real-time format using fluorescent probes. Results are obtained from unprocessed sputum samples in 90 min, with minimal biohazard and very little technical training is required. In our case, the solution collected after incubation of the sample in sterile distilled water was used as sputum equivalent and was decontaminated following Kent and Kubica method [14]. The decontaminated solution was mixed with the reagent that was provided with the assay, and a cartridge containing this mixture was placed in the GeneXpert machine. All the processes from this point on were fully automated [15].

### Statistical analysis

Descriptive statistics were used to establish the general characteristics of the study samples that were used to compare the bilateral Z test in Agricola Package in R software i386 3.2.2 (Vienna, Austria).

For each relative frequency P, a margin of error (ME) was calculated using the formula:


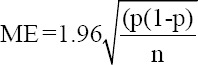


Where p is the relative frequency and n is the sample size [16] and the difference between frequencies was tested through Pearson’s Chi-squared test. The diagnostic and predictive value of the microscopic testing was assessed as described by [17]. The cycle threshold values represent the number of cycle at which the fluorescence intensity of each reaction intersects with the threshold generated automatically by the GeneXpert^®^.

## Results

### Macroscopic, microscopic, and molecular diagnosis

As shown in Table-1, TB lesions, mostly node lesions (95%) occurred most often in the lung (73%). About 65% of the samples collected were bovine TB positive based on the outcome of the molecular test, indicating a low rate of Mycobacterium DNA detection in most cases, while only 20% of the samples collected confirmed bovine TB positive based on the microscopic test (Table-1).

**Table-1:** Macroscopic, microscopic, and molecular diagnosis.

Variables	Level	Total number	Observed number	Frequency (%)
Type of lesion	Miliary lesion	40	2.00	5
	Nodes		38.00	95
Type of condemnation	Partial	40	40	100
Site of suspected lesions	Liver	40	3.00	7.5
	Maxillary muscle		1.00	2.5
	Esophagus		1.00	2.5
	Lungs		30.00	75
	Pre-scapular lymph node		1.00	2.5
	Retropharyngeal lymph node		4.00	10
Microscopy	Negative	40	32.00	80
	Positive		8.00	20
GeneXpert^®^ test	No	40	14.00	35
	Yes		26.00	65
Rate of detection	Low	26	14.00	54
	High		1.00	4
	Average		1.00	4
	Very low		10.00	38

### Comparison of microscopic and molecular diagnoses

The majority of bovine TB-negative samples based on the microscopic diagnosis were actually GeneXpert positive (62.5%). On the other hand, 25% of microscopy-positive samples were GeneXpert negative (Table-2). The sensitivity of the microscopic test was 23.08%; its specificity was 85.71%; while its positive predictive value was 75% and its negative predictive value was 37.50% (Table-3).

**Table-2:** Comparative analysis of microscopic and molecular test results.

Variable	Number	Frequency (%)
Microscopy+GeneXpert^®^ +	6	75
Microscopy+GeneXpert^®^ -	2	25
Microscopy - GeneXpert^®^ +	20	62.5
Microscopy - GeneXpert^®^ -	12	37.5

**Table-3:** Sensitivity, specificity, and predictive value of the microscopic test using GeneXpert® results as a reference.

Diagnosis test result	1 = GeneXpert^®^ +	2 = GeneXpert^®^ -	Total
A = Microscopy +	6	2	8
B = Microscopy -	20	12	32
Total	26	14	40

Sensitivity A1/(A1+B1) = 23.08% ; Specificity B2/(B2+A2) = 85.71%; Positive predictive value A1/(A1+A2) = 75.00%; Negative predictive value B2/(B2+B1) = 37.50%

### Incidence of TB in routinely examined slaughtered animals in relation to sex, breed, and age

Females (65%) are more affected than males (35%) and bovine TB incidence increased along with age, although the difference is not statistically significant (Table-4). White Fulani breeds are more affected than cattle from other breeds (p < 0.05).

**Table-4:** Bovine TB incidence according to gender, breed, and age.

Variables	Total number	Observed number	%	ME	p-value
Gender					
Female	26	17	65.38^a^	18.287	0.117
Male		9	34.62^a^	18.287	
Breed					
White Fulani	26	19	73.08^a^	17.050	0.047*
Borgou		1	3.85c	7.392	
Azawak		6	23.08^b^	16.195	
Age					
3	26	3	11.54^a^	12.281	0.3574
4		6	23.08^a^	16.20	
5		8	30.77^a^	17.74	
6		9	34.62^a^	18.29	

% Relative frequency, ME=Margin of error, TB=Tuberculosis. Frequencies within the column with the different letters are significantly (p < 0.05) different according to z-test

## Discussion

The outcome of our findings involves the study of bovine TB from suspected cases of the disease from postmortem examine slaughtered cattle and abattoir meat inspection of slaughtered cattle with the major aim of identifying how accurate is the currently used diagnostic method in Benin. Consequently, our results indicated that 35% of the bovine TB-like lesions at the slaughterhouses in Cotonou are due to non-tuberculous mycobacteria. Nuru *et al*. [18] have previously reported a more significant case of NTM species isolated from bovine TB-like lesions of grazing cattle slaughtered at Bahir Dar Abattoir in Ethiopia. Likewise, a study in Kenya reported a significant number of NTM isolates after culture and genotyping assay from suspected camels’ granulomatous lesions in slaughterhouses [19]. Our results indicated that the incidence of bovine TB was higher in cows than in bulls and in older animals than in younger ones. In fact, females are normally implicated as victims of bovine TB because they are normally kept for longer periods of time in the herd for the purpose of reproduction and milking, thereby increasing their chances of being infected. By implication, aged animals are normally prone to TB basically due to their low immune system. Such findings come in accordance with the previous study carried out by Agbalaya *et al*. [20]. This study also showed that White Fulani breeds of cattle have a higher incidence of coming down with TB than other breeds. However, this does not necessarily reflect a genetic predisposition of the breed to bovine TB. More comprehensive data over a longer period of time are therefore needed to shed light on the bovine TB susceptibility of cattle breeds inspected at the slaughterhouses.

The presence of node lesions, mainly in the lungs, retropharyngeal lymph nodes, and liver in our study corroborate with the previous work done in Bangladesh that showed that liver (50%) and lung (30%) were the most affected organs [21]. Sieng [22] has also previously reported that Bovine TB-like lymph nodes are mainly found around the head, neck, and chest.

Although culture was a reference method in the diagnosis of human TB, a variety of PCR methods have been developed recently and are reported to be more reliable, more sensitive, cost-effective, and time-saving [23]. Désiré *et al*. [24] showed the importance of real-time PCR in the epidemiologic diagnosis of resistance genes of *M. tuberculosis*. Joseph *et al*. [25] also reported that PCR assays are more accurate than single intradermal cervical tuberculin test. Sah *et al*. [26] have compared GeneXpert MTB/RIF assay and Multiplex PCR assay for direct detection of pulmonary TB in humans and they concluded that Multiplex PCR has higher sensitivity and specificity [26]. However, Carvalho *et al*. [27] have concluded that nested real-time PCR has higher efficiency when compared to multiplex PCR and microscopic tests in the detection of zoonotic TB. This could imply that the efficacy of the method used depends on the species in question.

The lower percentage of bovine TB detection through the microscopic test in our study confirms its low sensitivity, as suggested by Dubois [28]. With a positive predictive value of 75%, the microscopic test would only be effective if the samples examined had a high mycobacterium load. What explains the gap in between the molecular test (high sensitivity), up to 97.3%, no matter how low the bacterial load [15] and the microscopic test in our study. In another way, the microscopic test lack specificity, as some GeneXpert^®^ negative samples were microscopy positive. This is not surprising as bovine TB-like lesions could also be caused by other granuloma-forming organisms such as non-tuberculous mycobacteria and actinomycetes that happen to share the same tinctorial properties and thus are identified as acid-resistant bacilli by Ziehl–Neelsen staining [18, 29]. In human medicine, Mycobacterium other than *M. tuberculous* has been wrongly associated with TB [30]. Although their zoonotic potential is largely ignored [31], recently identified in Kenyan cattle non-tuberculous mycobacteria that have been linked to various types of mycobacteria in humans and concluded that zoonotic TB should not be restricted to *M. bovis* subsp. bovis.

This study has reiterated the endemicity of bovine TB in Benin, particularly at the studied slaughterhouses. It also informs of the limitation of the diagnostic tests currently used. The molecular test used in this study has proved to be more specific for bovine TB and, therefore can serve as a tool to strengthen the existing programs on the control and prevention of the disease to safeguard animal and human health. This will allow the collection of reliable data and better epidemiological surveillance of the disease. It is also important to investigate further the species involved in the bovine TB-suggestive lesions in Benin.

## Conclusion

This study address a One Health concept and highlight the importance of using molecular tools in the detection of Bovine TB. The application of Xpert is novel in human TB. However, it has never been used before in zoonotic TB in Benin. This study has reiterated the endemicity of bovine TB in Benin. It also informs of the limit of the diagnostic tests commonly used. The molecular test used in this study has proved to be more specific for bovine TB and, therefore can serve as a tool to strengthen the existing programs on the control and prevention of the disease. This will allow the collection of reliable data and better epidemiological surveillance of the disease.

## Authors’ Contributions

CKB, AZ, CMA, and HD: Conceptualization. HD, AZ, CMA, CKB, SF, and SM: Methodology. AZ and MM: Data collection and analysis. CKB and CMA: Supervision of the study. AZ, CKB, CMA, and SF: Writing-original draft. SM: Writing-review and editing. All authors have read and approved the final manuscript.
